# Effect of horizontal rectus surgery for the correction of intermittent exotropia on sub-A or sub-V pattern

**DOI:** 10.1371/journal.pone.0179626

**Published:** 2017-06-19

**Authors:** Young Bok Lee, Soolienah Rhiu, Joo Yeon Lee, Mi Young Choi, Hae Jung Paik, Key Hwan Lim, Dong Gyu Choi

**Affiliations:** 1 Department of Ophthalmology, Kangnam Sacred Heart Hospital, Hallym University College of Medicine, Seoul, Korea; 2 Department of Ophthalmology, Dongtan Sacred Heart Hospital, Hallym University College of Medicine, Dongtan, Korea; 3 Department of Ophthalmology, Hallym University Sacred Heart Hospital, Hallym University College of Medicine, Anyang, Korea; 4 Department of Ophthalmology, Chungbuk National University Hospital, Chungbuk National University College of Medicine, Cheongju, Korea; 5 Department of Ophthalmology, Gachon University Gil Medical Center, Gachon University School of Medicine, Incheon, Korea; 6 Department of Ophthalmology, Ewha Womans University Mokdong Hospital, Ewha Womans University School of Medicine, Seoul, Korea; Bascom Palmer Eye Institute, UNITED STATES

## Abstract

We evaluated effect of horizontal rectus surgery on sub-A or sub-V pattern intermittent exotropia. We enrolled patients with sub-A or sub-V pattern intermittent exotropia. The sub-A pattern was diagnosed when the eyes diverged less than 10 prism diopters (PD) from upgaze to downgaze, and sub-V pattern when the divergence was 14 PD or less from downgaze to upgaze. Patients had undergone horizontal rectus surgery without vertical transposition of horizontal rectus muscle or oblique muscle weakening. The patients were divided into two groups: sub-A pattern (group A) and sub-V pattern (group V). The outcome measures were change of amount of pattern and rate of collapse of pattern postoperatively. The amount of pattern (vertical incomitance) was amount of difference in exodeviation between upgaze and downgaze. Collapse of pattern was defined as disappearance of difference in exodeviation between upgaze and downgaze. In groups A and V, preoperative amounts of pattern were 4.9 PD and 6.8 PD, respectively. A significant reduction in amount of pattern was observed in both groups throughout the follow-up period (p<0.05). At postoperative 6 months, the amounts of pattern were 1.0 PD and 1.2 PD and the extents of reduction in amount of pattern were 4.4 PD and 5.9 PD. The rates of collapse of pattern at postoperative 6 months were 77.8 and 60.0%, respectively. In the patients with sub-A or sub-V pattern exotropia, horizontal rectus surgery without vertical transposition or oblique muscle weakening can successfully collapse the pattern.

## Introduction

The extent of deviation in patients with strabismus can change in upgaze and downgaze from the primary position, and sometimes increases or decreases with coexistent strabismus type. This has been deemed the A or V pattern [1]. The A pattern generally indicates a difference of exodeviation between upgaze and downgaze of more than 10 prism diopters (PD), and the V pattern, more than 15 PD [2]. Whereas these patterns are often the result of oblique muscle overaction or palsy, they also can be caused by ectopic muscle courses with ectopic pulleys, rotated orbit associated with craniofacial abnormalities, or nerve disdirection [1–5]. Shin and associates suggested that the A or V pattern in intermittent exotropia may come from the mechanical aspects of the oblique muscles. When the eye adopts a exotropic position, the oblique muscles become slack. Over time, they become shortened, causing tonic imbalance of the oblique muscles, when eventually may lead to the ocular torsion in primary position and pattern [6]. Additionally, Miller and Guyton demonstrated that loss of fusion in intermittent exotropia may induce the ocular torsion, thereby resulting in A or V pattern strabismus [7]. In general, when A or V pattern strabismus is presumed to be caused by oblique muscle overaction, the superior or inferior oblique muscles, respectively, are surgically weakened. When A or V pattern strabismus occurs in the absence of oblique muscle overaction, horizontal muscle transposition or the slanting technique were proposed [8–14].

In clinical settings, many patients have presented with only a small vertical incomitance of horizontal deviation that does not meet the criteria for the classic A or V pattern. In the present study, we defined this condition as sub-A or sub-V pattern strabismus. In a previous study, Lee et al. performed horizontal rectus surgery without oblique muscle weakening or horizontal muscle transposition for 25 patients with sub-V pattern exotropia [15]. They reported that 23 patients (92%) showed a successful surgical outcome (vertical incomitance of 5 PD or less). However, they did not evaluate the surgical outcomes of horizontal rectus surgery without oblique muscle weakening or horizontal muscle transposition for the patients with sub-A pattern exotropia. To the best of our knowledge, there has as yet been no investigation conducted to evaluate the efficacy of horizontal rectus surgery for correction of the sub-A pattern in intermittent exotropia. Therefore, we undertook to evaluate the effect of horizontal rectus surgery on the sub-A or sub-V pattern in intermittent exotropia.

## Materials and methods

We retrospectively reviewed the medical records of patients who had undergone horizontal rectus surgery for intermittent exotropia with the sub-A or sub-V pattern and been followed-up on postoperatively for 6 months or more. The sub-A pattern was diagnosed when the eyes diverged less than 10 prism diopters (PD) from upgaze to downgaze, and the sub-V pattern when 14 PD or less from downgaze to upgaze. Informed written consent for the surgical procedure had been obtained from all of the patients or their parents. Our study was approved by the Institutional Review Board of Hallym University Medical Center and was performed according to the tenets of the Declaration of Helsinki.

The patients were assigned to two groups: group A, those with sub-A pattern exotropia, and group V, those with sub-V pattern exotropia. The exclusion criteria were as follows: (1) combined surgery entailing vertical transposition of the horizontal rectus muscle or the oblique muscle weakening procedure for correction of the A or V pattern, (2) history of previous strabismus surgery, (3) trauma, (4) paralytic or restrictive exotropia, (5) other ocular disease, or (6) systemic disease such as Down syndrome or cerebral palsy.

### Preoperative examination

We noted preoperative characteristics including age at surgery, sex, mean angle of deviation at distance and near, stereopsis, refractive error, presence of vertical deviation, lateral incomitance, dissociated vertical deviation (DVD), amblyopia, and oblique muscle dysfunction. Vertical deviation was defined as 5 PD or more hypertropia/hypotropia at the primary position. Lateral incomitance was defined as a change of 5 PD or more in lateral gaze from the primary position. Amblyopia was defined as a between-eye best-corrected visual acuity difference of 2 lines or more. Oblique muscle dysfunction was graded on a 9-point scale, from +4 overaction to -4 underaction, with 0 being normal.

All of the patients underwent complete ophthalmologic examinations prior to the surgery. Visual acuity was measured on the Snellen visual acuity chart. Cycloplegic refraction, if needed, was performed with 1% cyclopentolate chloride. The angle of deviation was measured by alternate prism cover test at distance and near (6 m and 0.33 m) with best optical correction for all fields of gaze using accommodative targets. This measurement was double-checked by two different ophthalmologists (surgeon and assistant surgeon). If the exodeviation at distance was larger than 10 PD or comparable to that at near, we occluded one eye for 1 hour to eliminate fusional convergence, and then repeated the alternate prism cover test at distance and near. Measurement of the angle of deviation in upgaze and downgaze was performed by tilting the head approximately 25 degrees down and up, respectively. Stereopsis was examined by Titmus Stereotest (Stereo Optical Co., Inc., Chicago, IL, USA). Based on the degree of stereopsis, the subjects were divided into two groups: good (40–100 arcsec) and poor (>100 arcsec). Before disruption of fusion by alternate prism cover test, the Worth-4-dot test was performed at distance (6m), and the results were recorded as follows: (1) fusion and (2) no fusion, composed of suppression and diplopia.

### Strabismus surgery

Horizontal rectus surgery, including unilateral lateral rectus recession (ULR), bilateral LR recession (BLR), or unilateral recess-resect (R&R), was performed to correct the exodeviation at the primary position without a vertical transposition or oblique muscle weakening procedure. All of the surgeries were performed from January 2014 to February 2015 under general anesthesia according to the formula indicated on the surgical table suggested by Parks (based on the angle of exodeviation at distance) [16]. Unilateral LR recession and R&R were performed on the non-dominant eye.

### Postoperative management

The angle of deviation was measured at postoperative 1 day, 1 week, 1, 3, 6 months, and 1 year. On each visit, the subjective diplopia was recorded, and any abnormality in duction and version was examined. Alternate full-time patching was prescribed for the patients who had developed diplopia or esodeviation from postoperative 1 day, and was continued until the diplopia or esodeviation was resolved.

### Outcome measures

The outcome measures were the change of the amount of pattern and the rate of collapse of pattern postoperatively. The amount of pattern (vertical incomitance) was the amount of difference in exodeviation between upgaze and downgaze. The collapse of pattern was defined as the disappearance of difference in exodeviation between upgaze and downgaze.

### Statistical analysis

Statistical analyses were performed by an independent statistician. The statistical analysis was performed with SPSS software, version 21.0K (SPSS Inc., Chicago, IL, USA). The Paired t test and Wilcoxon signed rank test were used to compare the pre- and postoperative amounts of sub-A or sub-V pattern in each group. Statistical differences were considered significant when the P value was less than 0.05. Results are expressed as means ± standard deviations.

## Results

Fifty-eight patients who had undergone horizontal rectus surgery for sub-A or sub-V pattern intermittent exotropia were enrolled in this retrospective study. Among them, 12 patients had been diagnosed as sub-A pattern intermittent exotropia (group A) and 46 as sub-V pattern intermittent exotropia (group V). The horizontal rectus surgery was performed as follows: ULR in 7 patients (12.1%), BLR in 29 (50.0%), and R&R in 22 (37.9%) (Table 1). The mean age at surgery in groups A and V was 8.6 ± 1.8 years (range: 4.6–11.1) and 9.7 ± 5.4 years (range: 3.9–12.1), respectively. The postoperative mean follow-up period was 8.5 ± 4.5 months (range: 3–18) in group A and 8.2 ± 3.9 months (range: 3–12) in group V. The preoperative angle of exodeviation at distance was 27.7 ± 6.1 PD (range: 20–37) and 27.5 ± 7.5 PD (range: 16–45) in groups A and V, respectively. The preoperative amount of pattern was 4.9 ± 2.1 PD (range: 2–8) and 6.8 ± 4.1 PD (range: 2–12) in groups A and V, respectively. All patient characteristics are summarized in Table 2.

**Table 1:** Surgical procedures for intermittent exotropia.

Surgery	Group A (n = 12)	Group V (n = 46)
Unilateral LR recession (ULR)	0	7
Bilateral LR recession (BLR)	5	24
Unilateral recession-resection (R&R)	7	15

Group A = patients with sub-A pattern exotropia

Group V = patients with sub-V pattern exotropia

LR = lateral rectus muscle

**Table 2:** Preoperative demographic data.

	Group A (n = 12)	Group V (n = 46)
Age at surgery (years)	8.6 ± 1.8	9.7 ± 5.4
Sex (M/F)	6/6	28/18
Preoperative angle of exodeviation (PD)		
Distance	27.7 ± 6.0	27.5 ± 7.5
Near	28.7 ± 8.0	28.1 ± 10.6
Stereopsis (seconds of arc)		
Good (n, %)	9, 75.0%	30, 65.2%
Poor (n, %)	3, 25.0%	16, 34.8%
Fusion on W4D test at distance (n, %)	6, 50.0%	14, 30.4%
Refractive error (diopters)	-0.1 ± 1.4	-0.9 ± 1.9
Lateral incomitance (n, %)	1 (8.3%)	3 (6.5%)
Amblyopia (n, %)	1 (8.3%)	2 (4.3%)
Dissociated vertical deviation (n, %)	0 (0%)	2 (4.3%)
Vertical deviation (n, %)	3 (25.0%)	13 (28.3%)
Oblique muscle dysfunction (n, %)	2 (16.7%)	8 (17.4%)
Postoperative follow-up duration (months)	8.5 ± 4.5	8.2 ± 3.9

Group A = patients with sub-A pattern exotropia

Group V = patients with sub-V pattern exotropia

PD = prism diopters

Stereopsis: Good (40–100 arcsec), Poor (> 100 arcsec)

W4D = Worth-4-dot

Lateral incomitance = change of 5 PD or more in lateral gaze from primary position

Vertical deviation = 5 PD or more hypertropia/hypotropia at primary position

### Amount of sub-A or sub-V pattern (Tables 3 and 4)

**Table 3:** Amount of pattern (PD) in group A.

	Group A (n = 12)	P value^a^
Preoperative	4.9 ± 2.0	
Postoperative		
1 week	1.9 ± 2.3	0.022
1 month	1.2 ± 1.3	0.002
3 months	1.2 ± 1.9	0.005
6 months	1.0 ± 2.1	0.007

Amount of pattern = extent of difference in exodeviation between upgaze and downgaze

PD = prism diopters

Group A = patients with sub-A pattern exotropia

^a^Wilcoxon signed rank test for comparison between preoperative and postoperative variables

**Table 4:** Amount of pattern (PD) in group V.

	Group V (n = 46)	P value^a^
Preoperative	6.8 ± 4.1	
Postoperative		
1 week	1.8 ± 3.6	0.000
1 month	1.6 ± 2.2	0.000
3 months	1.0 ± 1.6	0.000
6 months	1.2 ± 1.9	0.000

Amount of pattern = extent of difference in exodeviation between upgaze and downgaze

PD = prism diopters

Group V = patients with sub-V pattern exotropia

^a^Paired t test for comparison between preoperative and postoperative variables

The preoperative amounts of pattern were 4.9 ± 2.1 PD in group A and 6.8 ± 4.1 PD in group V. The extent of reduction in the amount of pattern was 3.1 ± 3.4 PD in group A and 4.9 ± 5.1 PD in group V at postoperative 1 week. The reduction in the amount of pattern was maintained in both groups from postoperative 1 week to 6 months. At postoperative 6 months, the amounts of pattern were 1.0 ± 2.1 PD (range: 0–6) in group A and 1.2 ± 1.9 PD (range: 0–7) in group V, which were significantly different compared to preoperative amounts of pattern in both groups (group A, p = 0.007; group V, p = 0.000). And the extent of reduction in the amount of pattern was 4.4 ± 2.0 PD (range: 2–8) in group A and 5.9 ± 3.9 PD (range: 2–10) in group V.

### Rate of collapse of pattern

At postoperative 6 months, the rates of collapse of pattern were 77.8% in group A and 60.0% in group V, which suggested that many patients had gained complete remission of sub-A or sub-V pattern exotropia by horizontal rectus surgery only.

## Discussion

Different theories have been posited to explain the cause of the A or V pattern. Urist and Breinin both suggested horizontal rectus muscle abnormality [17,18]. Knapp and Breinin noted, in this regard, that contraction and relaxation of the horizontal rectus muscles occur at the midline upgaze and downgaze positions [13,19]. Scott reported that abnormal innervation to the LR muscle is the cause of divergence in upgaze in patients with V-pattern exotropia [20,21]. More recently, Jampolsky and Kushner established that oblique muscle dysfunction induces A- or V-pattern strabismus. Most notably, they associated inferior oblique muscle overaction with V-pattern strabismus, and superior oblique muscle overaction with A-pattern strabismus [1,3].

In the selection of the surgical technique for treatment of A- or V-pattern strabismus, the presence of oblique muscle dysfunction should be considered. When, for example, A- or V-pattern strabismus is caused by oblique muscle overaction, the superior or inferior oblique muscles are surgically weakened. Or, when A- or V-pattern strabismus occurs in the absence of oblique muscle overaction, horizontal muscle transposition or the slanting technique is performed [8–14].

In clinical settings, many patients present with a small vertical incomitance of horizontal deviation that does not meet the criteria of either the classic A or V pattern. In the present study, we enrolled patients with sub-A or sub-V pattern exotropia in order to evaluate the efficacy of horizontal rectus surgery for pattern correction. In a previous study, Lee et al. performed horizontal rectus surgery without oblique muscle weakening or horizontal muscle transposition for 25 patients with sub-V pattern exotropia [15]. They reported that 23 patients (92%) showed a successful surgical outcome (vertical incomitance of 5 PD or less). Also, they emphasized that the surgical success rate of oblique muscle weakening or horizontal muscle transposition did not differ from that of horizontal rectus surgery. In our study, the rate of collapse of pattern (60% of 46 patients with sub-V pattern exotropia at postoperative 6 months) was lower than reported by Lee et al. This discrepancy can be explained by the difference between Lee et al.’s definition of “collapse of pattern” and ours: “difference of 5 PD or less in exodeviation between upgaze and downgaze” and “disappearance of difference in exodeviation between upgaze and downgaze,” respectively.

To the best of our knowledge, there has as yet been no investigation conducted to evaluate the efficacy of horizontal rectus surgery for correction of the sub-A pattern in intermittent exotropia. In the present study, the amount of pattern and the extent of reduction in the amount of pattern at postoperative 6 months were 1.0 ± 2.1 PD and 4.4 ± 2.0 PD, respectively. The rate of collapse of pattern was 77.8%, most patients having experienced complete remission of the sub-A pattern. Considering these results, we concluded that sub-A pattern exotropia was successfully corrected by horizontal rectus surgery without the horizontal muscle transposition or oblique muscle weakening procedure.

Based on our findings, we wonder why the sub-A or V pattern gets smaller only after horizontal muscle surgery. In a previous study, Miller and Guyton demonstrated that loss of fusion in intermittent exotropia may induce the ocular torsion, thereby resulting in A or V pattern strabismus [7]. In other words, the status of fusion is instrumental in development of A or V patterns, consistent with sensory torsion theory of A and V pattern development. Generally, the sensory status in the patients with intermittent exotropia improved after surgery. In our study, it seems possible to presume that the change of sensory status after horizontal rectus surgery may affect the extent of A and V patterns. However, we could not explain the precise underlying mechanism. Thus, further research should be conducted to elucidate the underlying mechanism.

There are some limitations to our study. First, as it was a retrospective investigation, selection bias might have occurred. Therefore, the results will need to be confirmed through prospective study. Second, a relatively small number of patients with sub-A or V pattern exotropia were included. To extrapolate the results of this study on the efficacy of horizontal rectus surgery for correction of the sub-A or sub-V pattern and compare the efficacy of horizontal rectus surgery for the sub-A or sub-V pattern between the different types of horizontal rectus surgery (ULR, BLR, and R&R), then, a larger and randomized study should be conducted. Third, sub-A and V patterns were hard to measure accurately and even harder to measure when they get smaller after surgery. Although we double-checked the small A and V patterns by two ophthalmologists to improve the accuracy of measurement, the minor error may not be avoided completely. We believe that recording eye position using trackers in a far more rigorous fashion, as in future study, may eliminate the minor measurement error.

In summary, horizontal rectus surgery without horizontal muscle transposition or oblique muscle weakening presented favorable surgical results (collapse of pattern in 77.8 and 60% of sub-A and sub-V cases, respectively). Therefore, in cases of sub-A or sub-V pattern intermittent exotropia, horizontal rectus surgery may be performed without horizontal muscle transposition or oblique muscle weakening for successful correction.

## Supporting information

S1 Table Extent of reduction in amount of pattern (PD) in groups A and V. (DOCX)

S2 Table Rate of collapse of pattern (%) in groups A and V. (DOCX)
