# Supplementary material for: Effect of horizontal rectus surgery for the correction of intermittent exotropia on sub-A or sub-V pattern
Source: PLoS One. 2017 Jun 19;12(6):e0179626. doi: 10.1371/journal.pone.0179626 (PMC5476252; doi:10.1371/journal.pone.0179626)
Supplement: S1 Table — (DOCX) [file pone.0179626.s001.docx]

S1 Table. Extent of reduction in amount of pattern (PD) in groups A and V

| Postoperative | Group A (n=12) | Group V (n=46) |
| --- | --- | --- |
| 1 week | 3.0 ± 3.4 | 4.9 ± 5.1 |
| 1 month | 3.7 ± 1.9 | 5.2 ± 4.0 |
| 3 months | 3.9 ± 2.4 | 6.4 ± 3.9 |
| 6 months | 4.4 ± 2.0 | 5.9 ± 3.9 |

Amount of pattern = extent of difference in exodeviation between upgaze and downgaze

PD = prism diopters

Group A = patients with sub-A pattern exotropia

Group V = patients with sub-V pattern exotropia
