# Supplementary material for: Effect of horizontal rectus surgery for the correction of intermittent exotropia on sub-A or sub-V pattern
Source: PLoS One. 2017 Jun 19;12(6):e0179626. doi: 10.1371/journal.pone.0179626 (PMC5476252; doi:10.1371/journal.pone.0179626)
Supplement: S2 Table — (DOCX) [file pone.0179626.s002.docx]

S2 Table. Rate of collapse of pattern (%) in groups A and V

| Postoperative | Group A (n=12) | Group V (n=46) |
| --- | --- | --- |
| 1 week | 41.7% | 46.7% |
| 1 month | 41.7% | 48.8% |
| 3 months | 63.6% | 61.1% |
| 6 months | 77.8% | 60.0% |

Collapse of pattern = disappearance of difference in exodeviation between upgaze and downgaze

Group A = patients with sub-A pattern exotropia

Group V = patients with sub-V pattern exotropia
